# Surgeons and Damage Suits

**Published:** 1905-12

**Authors:** J. W. Kenney

**Affiliations:** San Antonio, Texas


### TEXAS MEDICAL JOURNAL.

ESTABLISHED JULY, 1885.

PUBLISHED MONTHLY.—SUBSCRIPTION $1.00 A YEAR.

VOL. XXI. AUSTIN, DECEMBER, 1905. No. 6.

The pnblisher is not responsible for the views of contributors.

For Texas Medical Journal.

### Surgeons and Damage Suits.

BY J. W. KENNEY, M. D., SAN ANTONIO, TEXAS.

Surgeons in their relation to personal injury damage suit litigation are divisible into three classes: (1) The Corporation Surgeon; (2) The Damage Suit Syndicate Surgeon; (3) The Independent Surgeon.

The corporation surgeon, unless he be a novice, realizes that the public in géneral is very charitable toward him. They believe that he may be honest, but if so, he is very ignorant.

The damage suit syndicate surgeon is likewise dealt with in a kindly manner by the laity. He is considered learned in his profession, but has unfortunately become a mere puppet in the hands of damage suit lawyers.

The independent surgeon stands aloof from such influences, and in his own estimation has a "holier than thou" appearance. To the interested, however, he is considered "on the fence," with a decided inclination to fall on either side when properly persuaded.

The corporation surgeon and the damage suit syndicate surgeon occupy the same position; their duties, however, are antagonistic. The former is supposedly employed to look after and care for the sick and injured employes of his master, while, in reality, he is an advance claim agent. The comfort and future welfare of his patient are as nothing compared to the securing of a patient's signature to a release that will legally prevent a suit for damages. So important are these stereotyped releases that they are included in all first-aid packages and have a secure place on all relief trains.

Hemorrhage, pain and undue mobility of bones are looked after, but with each single effort to correct these morbid phenomena there are a dozen to forestall a future damage suit. When called to attend the injured the corporation surgeon is provided with capable assistants, consisting usually of one medical assistant, one assistant claim agent, two or three detectives, and several disinterested witnesses. The doctor is armed with his emergency surgical paraphernalia, while his assistant, the claim agent, is armed with a large roll of "currency,"—the denomination of each bill growing smaller as it reaches the center of the roll,—which he handles in a very careless manner in the presence of the injured. Parenthetically, I may state that this is one mode of testing the eyesight that I have never seen mentioned in text-books. If the emergency work of the corporation surgeon fails in its main purpose, namely, getting the patient's signature to the release, he is not to lose heart. The hemorrhage, pain and broken bones are attended to and the patient conveyed to the hospital, which is provided by the company out of the wages of its employes. Into the sacred portals of this house none but corporation boosters are admitted without protest,—at least not until the release is signed. A patient who escapes from the seductive influence of such a combination is considered by the corporation surgeon to be in perfect physical and mental condition, and is so pronounced. In this opinion he is backed up by all his supernumeraries.

The damage suit syndicate surgeon, like his fraternal brother above mentioned, has duties that are not always presented to the public. Being selected on account of intellect, instead of pull, he usually starts out with a considerable advantage over his corporation brother. His duties, however, are, as the Irishman would say, "The same, only the reverse." The frequency with which a majority of those injured seek his advice after escaping from the corporation surgeon is truly wonderful,—the patient himself scarcely knowing how it happened. This surgeon is, likewise, provided with able assistants consisting of one medical assistant, one case finder, and several boosters, or ambulance chasers, thus named, perhaps, because they have contracted the habit of quickly locating the injured and giving them confidential advice as to the superiority of certain surgeons and the dangers of release signing. The confidence of the patient is thus shaken in his attending surgeon, and in his effort to become satisfied, he changes doctors, and very naturally falls into the hands of the damage suit syndicate surgeon, in whose presence all ills sticketh tighter than a brother. This surgeon's duty is not only to see that the patient does not get well, but, likewise, to guide him safely into the maw of the damage suit lawyer,— to teach the great therapeutic value of walking on crutches, and, finally, to demonstrate that the injuries are of such a nature as to totally disable the patient and prevent him enjoying life in this world or the world to come. In this opinion he is backed up by all his supernumeraries.

The independent surgeon doubtless tries to obey the dictates of his conscience while occupying his seat upon the fence. Being thus seated, however, and at the same time, human, he is easily seen and much sought after by both sides to the controversy. Moulding his opinion is a duty both the corporation and damage surgeons owe to their masters. That both are successful is self-evident from the expert testimony given in most all suits for damages due to personal injuries.

That this classification is unpleasant none will deny. That in so far as the public mind is concerned, it is a reality we all know. That more humiliating truths could be told many of us feel assured.

The unpleasant part of this classification is rendered more unpleasant when each member of our profession tries in vain to eliminate himself. The one making the loudest profession of innocence is, in all probability, the one who the public declares can give an opinion in a case as well before as after examining a case. In other words, that he is a one-sided man, and that his opinion is going to favor that side regardless of the patient's condition.

That more humiliating truths could be told is evidenced by the direful results following premature release signing, and the scientific manner in which many diseases are simulated.

The cause of the predicament in which we find ourselves is undoubtedly the worship of mankind for that uncrowned king of the universe—money. Relief is difficult to procure, for we can not remove the cause. Publicity of the methods persued by both sides would go a long ways toward bringing about the much-desired result. There would be very few sudden recoveries of permanently injured claimants after the receipt of the corporation treasurer's check, if the fact of the sudden recovery were published to the world. There would be less haste on the part of corporations to get the injured to sign a release if the picture of a totally disabled father in destitute circumstances with a large family on his hands could be held up to the public as a direct result of the hasty signing away of the father's legal right to indemnity upon the advice of a corporation surgeon that he would soon be well and able to resume his work.

If these every-day happenings could be presented to the public in their true light we would doubtless hear of cripples, upon their complete recovery, returning to the corporations the money mistakenly taken from them, and of corporations calling upon cripples whom they had mistakenly caused to sign away their rights to pecuniary damages and delivering up to them their just financial deserts.

